# Assessment Methods for Marginal and Internal Fit of Partial Crown Restorations: A Systematic Review

**DOI:** 10.3390/jcm12155048

**Published:** 2023-07-31

**Authors:** Adolfo Di Fiore, Andrea Zuccon, Filippo Carraro, Michele Basilicata, Patrizio Bollero, Giovanni Bruno, Edoardo Stellini

**Affiliations:** 1 Department of Neuroscience, Section of Prosthetic and Digital Dentistry, University of Padova, 35122 Padova, Italy; andrea.zuccon@unipd.it (A.Z.); giovanni.bruno.1@unipd.it (G.B.); edoardo.stellini@unipd.it (E.S.); 2 Department of Systems Medicine, University of Rome Tor Vergata, 00133 Roma, Italy; michele.basilicata@ptvonline.it (M.B.); patrizio.bollero@ptvonline.it (P.B.)

**Keywords:** inlay, marginal fit, internal fit, micro-CT, replica-technique

## Abstract

Background: Different methods are used for the analysis of marginal and internal fit of partial crowns, but not all of them are applicable for in vivo studies. The aim of this review is to search the available methods, described in the current literature, to assess marginal and internal fit in partial crowns. Methods: an electronic search was performed on Pubmed and Web of Science databases to find studies published from 1 January 2017 up to 2 March 2023, following PRISMA guidelines and Cochrane handbook for systematic reviews. The search strategy applied was: “(marginal) AND (fit OR gap OR adaptation OR discrepancy) AND (inlay OR onlay OR partial crown)”. In vitro studies which evaluated marginal and internal fit on CAD CAM or 3D printed partial crowns were included in this review. Quality of the studies was assessed by using Quality Assessment Tool For In Vitro Studies (QUIN tool). Results: 22 studies were included. Among conventional methods, direct view with microscope, indirect view on resin replicas, and silicone replica technique (SRT) were used. Considering new digital methods, micro-CT, SRT 3D and triple scan technique (TST) were applied. Conclusions: Among 2D methods, direct view technique is the most used marginal fit analysis. For a more comprehensive evaluation, a 3D digital analysis is suggested. SRT and indirect view are the only 2D methods available for in vivo analysis. A protocol for the application of TST for assessment in vivo is now available, but no studies are reported in literature yet.

## 1. Introduction

In the field of restorative dentistry, partial crowns represent an important choice for dental treatments. This kind of indirect restoration can be preferred by the clinician in case of medium to large cavities, when polymerization shrinkage of direct composite restorations can negatively affect the long-term prognosis of the teeth [1,2]. Moreover, partial crowns are less invasive compared to full crowns in terms of preparation of the cavity and remaining dental tissues [3]. In literature, their optimal aesthetic properties and durability are reported by many studies [4,5,6,7,8]. Nowadays, thanks to the development of CAD-CAM technologies, the chairside production of indirect restoration is very common [9,10]. This process consists firstly of an intraoral scanner to obtain the digital impression in the oral cavity and a digital software for data analysis and restoration design [11]. Secondly, a subtractive or additive manufacturing device is necessary to proceed on the realization of the prothesis [12,13,14]. This digital workflow offers many advantages to clinicians and patients, being a more time-saving technique and guaranteeing a more standardized process [15]. When a dental treatment for partial crowns is planned, one of the most important parameters that must be considered is represented by marginal and internal fit. Holmes defines the internal gap as the perpendicular measurement from the internal surface of the casting to the axial wall of the preparation, and the marginal gap as the same measurement at the margin [16]. Poor marginal fit decreases the long-term durability of the restorations, leading to microleakage, secondary caries, gingival inflammation, and cement dissolution, while insufficient internal fit increases the risk of fracture of the prothesis [17,18]. For marginal and internal fit evaluation, different methods are available, and they are distinguished mainly as destructive or non-destructive and 2D or 3D methods [19,20]. The existence of a systematic review that takes in consideration the description of the different methods applied for the assessment of marginal and internal fit on partial crowns is unknown to the authors. Moreover, in literature there are very few studies that analyse marginal and internal adaptation of partial crowns in vivo. 

The purpose of this systematic review is to recognize different methods used for the analysis of marginal and internal fit of partial crowns and to investigate which of these can be used for in vivo analysis.

## 2. Materials and Methods

This systematic review was conducted following the PRISMA guidelines (Preferred Reporting Items for Systematic review and Meta-Analysis) and Cochrane Handbook for systematic reviews [21].

A PICO strategy was established to help formulate the main question:

P: partial crowns, inlays, onlays, overlays

I: assessment methods for analysis of the outcome

C: not applicable

O: marginal and internal fit

The main question stated was the following: “what are the available methods described in literature for the evaluation of marginal and internal fit of inlays, onlays and partial crowns produced following a digital workflow?” A secondary question was formulated: “which of these methods could be used for analysis of marginal and internal fit in clinical studies?”. The secondary and quantitative outcome is the number of points assessed per specimen and mean values of marginal and internal fit.

### 2.1. Search Strategy

An electronic search was conducted on Pubmed and Web of Science to find relevant publications about this topic. The search strategy used was the following: marginal AND (fit OR gap OR adaptation OR discrepancy) AND (inlay OR onlay OR partial crown). Time restrictions were applied, considering studies published from 1 January 2017 to 2 March 2023. No language restrictions were taken in consideration. At the end, manual search was carried out by consulting references of the selected studies.

### 2.2. Eligibility Criteria

#### 2.2.1. Inclusion Criteria

The criteria for the inclusion of the studies are the following: in vitro studies on CAD-CAM or 3D printed partial crowns, such as inlay, onlay or overlay, produced from cavity preparations made on human or model posterior teeth (molars and premolars). These studies must take in consideration the quantitative evaluation of marginal fit, and possibly of internal fit, expressed in micrometers or in percentage values of continuous margin. 

#### 2.2.2. Exclusion Criteria

For the exclusion of the studies the following criteria were considered: clinical studies, because, based on a preliminary search, only a few publications are available in literature that are focused on fit evaluation of partial crowns; literature reviews, books, abstract with no full text, papers, pilot studies, case reports; studies which consider only prosthetic veneers, full crowns, multiple unit bridges, implants, interim restorations and wax models; studies which evaluate only internal fit; partial crown restorations produced with conventional methods.

### 2.3. Studies Selection

In a first phase, after removing duplicates, two reviewers independently performed an accurate analysis of the titles and abstracts of the articles that emerged from the research on the electronic databases. To calibrate inter-examiner reproducibility, the following method was used: in case of disagreement regarding the inclusion of a study, the two authors discussed and reached a mutual consensus before coming to a final decision.

Then, full texts of the remaining articles were examined for the final selection. Finally, manual searching by reading references of selected articles was conducted. 

### 2.4. Data Extraction

The following data were extracted for table synthesis: first author, year of publication, type and material of restoration, number of specimens considered for fit evaluation, type of software and CAM technology used for restorations, selected cement space, parameters evaluated by the study (marginal fit/internal fit), marginal and internal fit evaluation method(s) applied, cementation, thermal cycling or thermomechanical loading, number of points assessed per specimen, mean or median values and standard deviation of marginal and internal fit. Data were extracted to assess the main outcome, that is represented by the method(s) applied for marginal and internal fit evaluation. Secondary outcomes are the following: number of points assessed per specimen and mean values of marginal and internal fit, expressed in micrometers or percentage of continuous margin. 

The degree of heterogeneity between the studies was too high to allow meta-analysis evaluation.

### 2.5. Quality Assessment

Quality of the selected studies was individually assessed. In accordance with the Quality Assessment Tool For In Vitro Studies (QUIN Tool), twelve different criteria were considered, which are the following: clearly stated aims/objectives, detailed explanation of sample size calculation, detailed explanation of sampling technique, details of comparison group, detailed explanation of methodology, operator details, randomization, method of measurement of outcome, outcome assessor details, blinding, statistical analysis, presentation of results [22]. Each criteria can be adequately specified (score = 2), not adequately specified (score = 1), not specified (score = 0) or not applicable (NA). Then, the twelve scores are added to obtain the final score for each study. At the end, the result obtained is used to grade each single study as high, medium, or low risk (>70% = low risk of bias, 50% to 70% = medium risk of bias, and <50% = high risk of bias) by using the following formula: Final score = (Total score × 100)/(2 × number of criteria applicable).

## 3. Results

This section may be divided by subheadings. It should provide a concise and precise description of the experimental results, their interpretation, as well as the experimental conclusions that can be drawn.

### 3.1. Studies Selection

**Figure 1:**
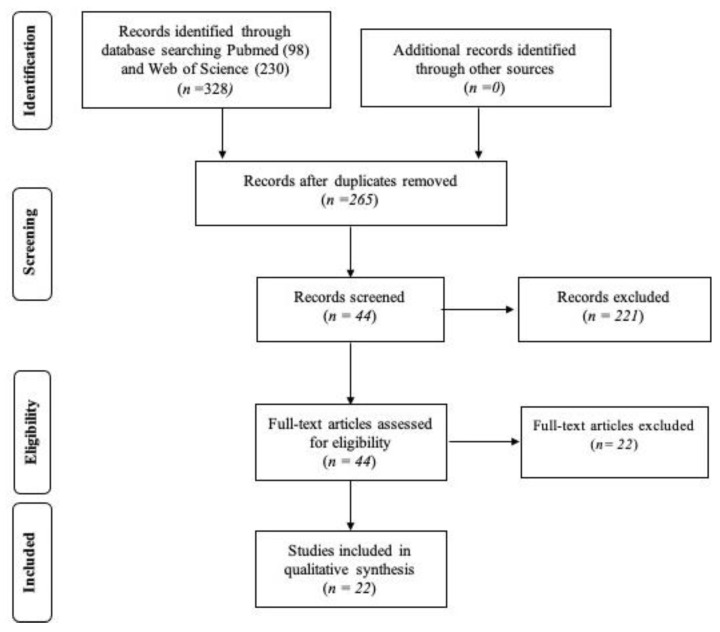
PRISMA flow chart of screened, withdrawn and included articles through the review process.

328 publications were found by electronic searching. After removing duplicates, 265 articles were obtained and 221 of them were excluded by reading title and abstract. 44 studies remained for full text examination. After this phase, 22 articles were included in the final review. The reasons for the exclusion of studies during this last analysis were the following: evaluation of internal fit only [23,24], pilot studies [25], case reports [26], reviews [20,27,28], in vivo studies [29], publication before 1 January 2017 [30,31,32,33], quantitative analysis of fit not expressed in micrometers or percentage values of continuous margin [34,35,36], analysis on wax models [37] or restorations produced with conventional methods [38,39,40,41,42,43] Figure 1.

### 3.2. Description of the Studies

The characteristics of the studies are reported and summarized on Table 1. For the analysis of marginal fit, the following methods were applied:

- direct view with optical or electronic scanning, used by eleven studies [44,45,46,47,48,49,50,51,52,53,54];
- indirect view on epoxy resin replicas, used by two studies [55,56];
- silicone replica technique and optical scanning, used by four studies [57,58,59,60];
- micro-CT analysis, used by two studies [61,62];
- silicone replica technique and 3D analysis, used by three studies [63,64];
- triple scan technique, used by one study [65].
  Internal fit was assessed by 9 studies using one of the following methods:
  - silicone replica technique with 2D optical scanning or 3D analysis
  - [46,57,59,63,64];
  - micro-CT [61,62];
  - triple scan technique [65].

**Table 1:** Data collection.

Author and Year of Publication	Type of Restoration and Materials	N Specimens Analysed	Points Measured per Specimen	Marginal Fit Values	Internal Fit Values
Lima et al., 2018 [44]	Onlay (2 design) RNC (Lava Ultimate)	40	18 (x3)	DDI: from 60 ± 39 to 71 ± 64 micron IDI: from 42 ± 33 to 75 ± 47 micron	/
Oz et al., 2018 [48]	MOD Inlay EC: IPS e.max CAD LU: RNC (Lava Ultimate) EL: IPS Empress CAD	45	12	EC: 33.54 ± 15.83 LU: 33.77 ± 17.35 EL: 34.23 ± 17.67 micron	/
Gudugunta et al., 2019 [51]	MOD Onlay IPS e.max CAD	15	60	41.46 ± 15.94 micron	/
Hamid et al., 2019 [47]	MOD Onlay RNC (Lava Ultimate)	12	10	From 27.81 ± 12.62 to 93.79 ± 17.97 micron	/
Neto et al., 2019 [46]	Onlay IPS e.max CAD	40	MF:16 (x3) IF:21	CO: 55.26 ± 46.85 CB: 41.70 ± 40.83 micron	CO: 161.13 ± 87.86 CB: 167.47 ± 92.04 micron
Qian et al., 2020 [50]	MOD Inlay EN: Vita Enamic LU: Lava Ultimate	24	8	Before TC: EN: 100.49 ± 32.03 micron LU: 91.19 ± 29.77 micron After 10.000 TC: EN: 105.79 ± 34.20 micron LU: 94.99 ± 32.78 micron	/
Falanchai et al., 2020 [45]	Overlay (4 designs) ZLS (Vita Suprinity)	40	20 (x3)	MF1: from 71.59 ± 14.60 to 91.66 ± 8.06 micron MF2: from 108.84 ± 13.68 to 128.31 ± 10.52 micron	/
Alenezi et al., 2021 [49]	Onlay e. max CAD	20	6	From 59 to 84 micron	/
Merrill et al., 2021 [60]	Inlay and onlay Feldspathic ceramic (Vita MkII)	40	n.d.	CO Inlay: 75.1 ± 7.1 micron CB Inlay: 116.2 ± 29.0 micron CO Onlay: da 104.1 ± 34.3 micron CB Onlay: 133.3 ± 38.5 micron	/
Rippe et al., 2016/17 [57]	MOD Inlay -Re: RC (Lava Ultimate) -Dis: e.max CAD (Ivoclar Vivadent)	30	MF: 6 (x3) IF: 19 (x3)	LaRe: from 105.9 ± 40.3 to 130.9 ± 38.4 micron CeRe: from 116.7 ± 42.1 to 145.3 ± 106.5 micron CeDis: from 171.8 ± 56.6 to 177.8 ± 68.9 micron	LaRe: from 104.7 ± 13.9 to 233.8 ± 80.5 micron CeRe: from 76.7 ± 24.6 to 227.5 ± 94.2 micron CeDis: from 66.7 ± 19.9 to 207.2 ± 61.3 micron
Sharma et al., 2020 [58]	MOD Inlay Zirconia (Cercon HT)	30	7 (x2)	From 20.16 ± 1.55 to 40.43 ± 1.27 micron	/
Lim et al., 2023 [59]	Inlay -LU: Lava Ultimate, 3M Espe -ZR: Zolid Fx multilayer -3D: Nextdent C&B	39	MF: 2 IF: 4	LU: 118.54 ± 45.54 micron ZR: 58.35 ± 14.88 micron 3D: 53.77 ± 16.29 micron	LU: 168.81 ± 42.67 micron ZR: 95.69 ± 13.34 micron 3D: 82.02 ± 8.32 micron
Negucioiu et al., 2019 [54]	Onlay HC (Vita Enamic) IPS Empress CAD	12	4	EN: from 88.10 ± 47.51 to 168.11 ± 79.71 micron IPS: from 72.70 ± 21.41 to 140.60 ± 142.53 micron	/
Frankenberger et al., 2021 [56]	MOD Onlay EM: e.max CAD (Ivoclar Vivadent) CD: Celtra Duo (Dentsply Sirona) ZR: Cercon Ht (Dentsply Sirona)	24	/	EM: from 95 ± 7 to 100% CD: from 93 ± 9 to 100% ZI: from 76 ± 23 to 100%	/
Soliman et al., 2022 [55]	Partial crown Celtra Duo (Dentsply sirona)	48	/	HV: from 267.71 ± 134.14 to 332.71 ± 175.16 micron LV: from 146.75 ± 71.73 to 165.98 ± 83.97 micron	/
Daher et al., 2022 [53]	Onlay MCOMP: Tetric CAD EM: IPS E.max CAD 3D: VarseoSmile Crown Plus	24	/	MCOMP: from 75.9 to 68.5% EM: from 63.1 to 43.7% 3D: from 69.8 to 44.7%	/
Bayrak et al., 2021 [61]	Onlay Feldspathic ceramic (Vita Enamic)	33	MF: 4 IF: 7	CE: from 48.8 ± 0.07 to 272.2 ± 0.11 micron Ka: from 76.0 ± 0.10 to 192.4 ± 0.13 micron Pl: from 90.2 ± 0 to 138.1 ± 0.11 micron	CE: from 77.0 ± 0.04 to 248.1 ± 0.07 micron Ka: from 63.2 ± 0.11 to 232.4 ± 0.10 micron Pl: from 72. 0 ± 0.02 to 278.4 ± 0.15 micron
Ekici et al., 2021 [62]	Inlay Feldspathic ceramic (CEREC Blocs)	36	MF: 2 IF: 5	CO: from 120.37 ± 84.82 to 121.51 ± 61.10 micron CB: from 16.05 ± 33.27 to 84.47 ± 30.04 micron CI: from 73.93 ± 112,20 to 83.77 ± 16.49 micron	CO: from 91.45 ± 44.93 to 184.33 ± 74.23 micron CB: from 23.36 ± 43.54 to 138.57 ± 52.29 micron CI: from 33.37 ± 53.21 to 179.71 ± 87.75 micron
Zimmermann et al., 2018 [64]	Inlay ZLS Celtra Duo (Dentsply Sirona)	30	MF: 1 ROI (region of interest) IF:2 ROI 20.000 points per surface	Group 12: 120.4 ± 12.9 micron Group 12 two step: 110.3 ± 22.2 micron Group 12s: 144.6 ± 144 micron	Group 12: from 96.9 ± 12.0 to 215.8 ± 14.4 micron Group 12 two step: from 90.5 ± 20.1 to 155.0 ± 40.1 micron Group 12s: from 122.8 ± 12.2 to 222.8 ± 35.6 micron
Yang et al., 2019 [63]	MOD Onlay (2 design) Ceramic reinforced composite resin (Hyramic, Upcera)	30	MF: 40 IF: 60	CP: from 47.1 ± 1.0 to 49.7 ± 1.4 micron SP: from 133.4 ± 1.1 to 135.8 ± 2.2 micron	CP: 51.8 ± 0.6 micron SP: 141.5 ± 8.1 micron
Kassis et al., 2021 [65]	MOD Overlay HT-14L: nano ceramic (Cerasmart) ZLS14 (Vita Suprinity)	30	MF: 5 IF: 6	HT-14L: 100.02 ± 19.60 micron ZLS14: 114.49 ± 21.50 micron	HT-14L: from 110.70 ± 13.91 to 118.68 ± 9.03 micron ZLS14: from 114.33 ± 18.14 to 137.00 ± 8.61 micron
Qian et al., 2022 [52]	22		MF: 8 IF: 2 ROI (region of interest)	3D Analysis group IDI: 119.32 ± 44.35 micron group DDI: 75.41 ± 8.66 micron 2D Analysis Before TC group IDI: 111.45 ± 33.97 micron group DDI: 74.43 ± 8.25 micron After TC group IDI: 124.77 ± 34.47 micron group DDI: 84.07 ± 7.31 micron	3D Analysis group IDI: 100.96 ± 22.53 micron group DDI: 72.05 ± 8.16 micron

Legend: RNC: resin nano-ceramic; DDI: direct digital impression; IDI: indirect digital impression; MOD: mesio-occluso-distal; CO: Cerec Omnicam scanner; CB: Cerec Bluecam scanner; CI: inEOS X5 scanner; La: Lava COS scanner; Ce: CEREC CAD System; Ka: Kavo CAD System; Pl: Planmeca CAD System; Re: composite resin; Dis: lithium disilicate ceramic; CP: conventional preparation; SP: shoulder preparation; HV: high viscosity composites; LV: low viscosity composites; MF: Marginal fit; IF: Internal fit; ROI: region of interest; TC: thermal cycling.

The number of points measured varied in relation to different method applied, as it is shown on Table 1. Most of the methods assessed marginal and internal fit by measuring the linear distance from single points, others considered whole regions of interest. Considering the studies where it was specified, the number of points assessed varied from 4 to 100. One study was able to evaluate 20.000 different points per surface during its analysis [64]. Twenty studies assessed quantitative values in micrometers. Marginal fit mean values ranged from 16 μm to 332.7 μm, while internal fit values ranged from 23.3 μm to 278.4 μm. Two studies evaluated marginal adaptation as the percentage of continuous margins (%CM), and values ranged from 63.1% to 100% before thermomechanical cycling, and from 43.7% to 95% after thermomechanical cycling.

### 3.3. Quality Assessment

The risk of bias of included studies is summarized in Table 2. All studies presented low to medium risk of bias, except for one that presented high risk of bias [54]. 

## 4. Discussion

This systematic review highlighted that different methods are available for the evaluation of marginal fit, while just some of them allow the analysis of internal fit. The most used method in the included publications is represented by the direct view analysis, that allows a more practical and non-destructive 2D measurement. It consists in using a standard or digital optical microscope, a stereomicroscope, or an electron microscope (SEM), for direct viewing of the selected points and for calculating linear distances between them. SEM evaluation is more detailed than an optical microscope, because of its higher resolution degree that varies from 100× to 1000×. Moreover, it is applicable also for indirect view analysis, as shown by Soliman et al. and Frankenberger et al. [55,56]. This non-invasive strategy aims to measure marginal fit on epoxy resin replicas of the specimens after having sputter-coated them with gold. In addition, Daher et al. and Frankenberger et al. used SEM to conduct a quantitative analysis of the integrity of the interface margins, by calculating the percentage of continuous margin, which refers to the relation between “gap-free” and “gap irregularity” on the whole margin assessed by the operator [53,56]. Considering direct view technique, instruments used are not suitable for in vivo analysis of fit. Then, none of the studies that used optical or SEM analysis measured the internal fit, because the specimen needs to be sectioned for its evaluation and consequently this would represent a destructive process. For this reason, silicone replica technique can be considered an alternative choice for 2D analysis of both marginal and internal fit. This method consists in the injection of a light-body silicone into the cavity of the tooth, simulating the cement layer, and the application of the specimen over the prepared tooth. This process is completed by using finger pressure or a testing machine. After the polymerization of light-body material the restoration is removed and a medium- or hard-body silicone is used for stabilization of the silicone specimen. In the end the silicone, which represents the space between tooth and restoration surfaces, is removed and sectioned for fit analysis through an optical microscope, as shown by the authors [57,58,59]. The study of Merrill et al. is the only one that evaluated marginal fit by not removing the layer of silicone, leaving the partial crown over the abutment tooth and consequently did not consider internal fit analysis [60]. These 2D methods represent an easy, not expensive, and repeatable way to measure directly or indirectly the adaptation on partial crowns in different points, which varied from 2 to 60 in the included studies. Replica silicone technique makes it simple to produce two or three replicas per specimen, obtaining a more consistent number of measurable points, as shown by Rippe et al. and Sharma et al. [57,58]. It is a simple, cheap and non-destructive technique. Its main disadvantage is represented by the low number of sections that can be cut and the risk of distortion of the material during this process, which can compromise the results of measurements. Then, a new digital and 3D analysis evaluation by using silicone replica technique is described, which consists of scanning firstly the abutment tooth and secondly the silicone layer over the abutment tooth. This strategy does not need the section of the material and allows to obtain a more comprehensive evaluation of marginal and internal fit by measuring hundreds to thousands of points or by calculating the mean gap in specific areas of interest. A digital software is necessary for the alignment of the scan data and for calculating marginal and internal gap. Yang et al. used Geomagic Control X software and selected 100 different points on the surface area (40 for marginal fit, 60 for internal fit), then aligned the two STL data and calculated the deviation between every point on the paired casts [63]. Qian et al. used Geomagic Qualify 12 to get the best fit alignment of the two data and calculated the root mean square for the whole internal surface by considering two different regions of interest, axial and pulp walls. The marginal fit was evaluated in 8 different regions [52]. Zimmerman et al., using Oracheck software, firstly superimposed the two files through best fit alignment, then selected three different areas of interest and matched about 20.000 points per surface, calculating the distances through software algorithm [64]. Bayrak et al. and Ekici et al. evaluated marginal and internal fit by using micro-CT [61,62]. It consists of a high-resolution radiological scanning device that reconstructs 2D and 3D digital images of the specimen on specific software, without being destructive. Both the authors used 2D section images on coronal and sagittal plane to assess linear distances from pre-selected points. Number of points assessed per specimen varied from 2 to 4 for marginal fit, and from 5 to 7 for internal fit. In addition, both authors performed a volumetric analysis, by using 3D reconstructed images to support 2D linear measurement. Bayrak et al. concluded that volumetric measurement can be relevant just to support 2D analysis, which is more important than 3D evaluation for final considerations. Micro-CT represent a non-destructive and precise method, although it cannot be used on patient due to its invasiveness. Furthermore, it is a more expensive technique, considering the costs of the instruments that are needed. The last digital method described is represented by the triple scan technique, firstly illustrated by Holst et al. [66]. Using an intraoral or a laboratory scanner, three scan data are achieved: data of the abutment tooth, data of partial crown restoration and finally the data of the partial crown positioned on the abutment tooth. Then a software makes it possible to obtain a best fit alignment between the first and the second data, for final analysis. Kassis et al. [65], in the study included in this review, used a Trios (3 Shape) scanner and Exocad software program to do quantitative analysis of marginal and internal fit. For each specimen, reference best-fit alignment function made it possible to standardize the reposition of scan data, and the distances between the tooth and the intaglio surface of the specimen were calculated in 11 different points as linear measurements. This technique is the only known strategy to conduct a full digital analysis of the adaptation of the partial crown. 

The number of points measured is an important factor to give consistency to the results. Groten et al. [67] concluded that a minimum of 50 measured points per specimen are necessary to obtain a more consistent estimate of the gap between abutment tooth and margin of the restoration. By the data obtained on Table 1, most of the studies included in the review did not fulfill this task. 2D analysis by direct or indirect view with optical or electronic microscope and conventional silicone replica technique do not allow to easily measure a consistent number of points per specimen. For this reason, some authors repeated two or three times the measurements on the same points to obtain more data. Micro-CT analysis was used for the assessment of marginal and internal gap after scanning the specimens by section data obtained. A 2D analysis was carried out, but an inconsistent number of points was assessed by both the authors. 3D analysis by using digital scanning devices and software is the only method that made it possible to measure easily more than 50 points, by selecting a more consistent number of points for linear measurements, or by using the best fit alignment of the scan data and considering different regions of interest for fit assessment, as shown by Yang et al. [63], Qian et al. [52], and Zimmerman et al. [64].

Most of the authors believe that the clinical acceptable limit for marginal fit is around 120 microns, as suggested in a first moment by Mclean and Franuhofer [68], instead, others consider values till 200 microns [60,61]. From data reported in Table 1, not all the studies reproduced mean values of marginal and internal fit within these limits. As reported by previous studies, many factors can affect these values, such as different CAD-CAM system used, material, design of preparation, pre-selected luting space, cementation of the restoration, thermal or thermomechanical loading. The heterogeneity of these parameters that characterize each study makes it difficult to compare the results of marginal and internal fit between them [20]. In vitro studies are useful to simulate circumstances of in vivo studies, but do not fully represent the conditions that distinguish the oral environment. In literature, few studies on partial crown are available for in vivo evaluation [26,29]. Among these methods, 2D analysis in vivo is possible using silicone replica technique or epoxy resin replicas, which represent the only non-invasive methods for patients. As seen in this review, methods that use 3D analysis protocol can give a more comprehensive evaluation of fit, by measuring a more consistent number of points and being more precise thanks to digital calculation of distances. Triple scan technique is the most promising method that allows the clinician to calculate marginal and internal fit using a full digital analysis, with the possibility to be used in the oral cavity of the patient. In literature, a new protocol is explained for its application in vivo, but no studies that use this protocol in clinical practice on partial crown are described [69]. So, it would be a new challenge to use this protocol for marginal and internal fit assessment on partial crown.

This review also presents some limitations. Firstly, there is a limited number of studies utilizing advanced 3D analysis methods, which offer a more comprehensive assessment by measuring a consistent number of points with higher precision. The scarcity of such research could be attributed to factors like the novelty and cost of the required technology. Secondly, the absence of standardized evaluation criteria across studies complicates comparisons and hinders drawing conclusive findings. Additionally, the commonly used direct view analysis, relying on optical or electron microscopes, introduces subjectivity due to human judgment, potentially affecting the reliability of measurements. Addressing these limitations would be crucial in advancing the understanding and application of partial crown fit assessment, leading to more accurate and consistent outcomes in clinical practice.

## 5. Conclusions

Based on the results found by this systematic review the following conclusion can be drawn:

- direct view method is the most common technique used for fit evaluation on partial crown, but 2D analysis does not allow a properly evaluation of the whole specimen;
- for in vivo analysis, silicone replica technique and indirect view with SEM can be applied;
- methods that are available for 3D analysis, such as silicone replica technique and triple scan protocol, permit to obtain more consistent data;
- triple scan technique represents a new full digital protocol for 3D analysis of fit in partial crowns, with promising characteristics that makes it suitable for clinical evaluation.

## Figures and Tables

**Table 2:** Risk of Bias assessment. Quin Tool Method.

	Clearly Stated Aims/Objectives	Detailed Explanation of Sample Size Calculation	Detailed Explanation of Sampling Technique	Details of Comparison Group	Detailed of Methodology	Ope Rtor Details	Randomization	Method of Measurement of Outcome	Outcome Assessor Details	Blinding	Statistical Analysis	Presentation of Results	SCORE	BIAS EVALUATION (Score x 100/2 x Number of Criteria Applicable)
Lima et al. [44]	2	0	0	2	2	0	0	2	0	0	2	**2**	**12**	**50.0%** **Medium risk**
Oz et al. [48]	2	0	2	2	2	0	2	2	1	0	2	**2**	**17**	**70.8%** **Low risk**
Gudugunta et al. [51]	2	0	0	2	2	1	0	2	1	0	2	**2**	**14**	**58.3%** **Medium risk**
Hamid et al. [47]	2	0	1	2	2	1	0	2	0	0	2	**2**	**14**	**58.3%** **Medium risk**
Neto et al. [46]	2	0	0	2	2	1	0	2	0	0	2	**2**	**13**	**54.2%** **Medium risk**
Qian et al., 2020 [50]	2	0	1	2	2	1	0	2	1	0	2	**2**	**15**	**62.5%** **Medium risk**
Falanchai et al. [45]	2	0	2	2	2	2	0	2	0	0	2	**2**	**16**	**66.7%** **Moderate risk**
Alenezi et al. [49]	2	0	0	2	2	0	0	2	0	0	2	**2**	**12**	**50.0%** **Medium risk**
Merrill et al. [60]	2	0	0	2	2	0	0	2	2	0	2	**2**	**14**	**58.3%** **Medium risk**
Rippe et al. [57]	2	2	2	2	2	0	2	2	0	0	2	**2**	**18**	**75.0%** **Low risk**
Sharma et al. [58]	2	0	0	2	2	1	0	2	1	0	2	**2**	**17**	**70.8%** **Low risk**
Lim et al. [59]	2	0	0	2	2	0	0	2	0	0	2	**2**	**12**	**50.0%** **Medium risk**
Negucioiu et al. [54]	2	0	0	2	2	1	0	2	0	0	0	**2**	**11**	**45.8%** **High risk**
Frankenberger et al. [56]	2	1	2	2	2	0	0	2	2	2	2	**2**	**19**	**79.2%** **Low risk**
Soliman et al. [55]	2	0	1	2	2	0	0	2	0	0	2	**2**	**13**	**54.2%** **Medium risk**
Daher et al. [53]	2	0	0	2	2	1	0	2	0	0	2	**2**	**13**	**54.2%** **Medium risk**
Bayrak et al. [61]	2	0	0	2	2	1	0	2	0	0	2	**2**	**13**	**54.2%** **Medium risk**
Ekici et al. [62]	2	0	2	2	2	1	0	2	0	0	2	**2**	**15**	**62.5%** **Medium risk**
Zimmermann et al. [64]	2	0	0	2	2	0	0	2	0	0	2	**2**	**12**	**50.0%** **Medium risk**
Yang et al. [63]	2	0	0	2	2	1	0	2	1	0	2	**2**	**14**	**58.3%** **Medium risk**
Kassis et al. [65]	2	2	1	2	2	1	0	2	1	0	2	**2**	**17**	**70.8%** **Low risk**
Qian et al., 2022 [52]	2	0	0	2	2	0	0	2	0	0	2	**2**	**12**	**50.0%** **Medium risk**

SCORE: adequately specified: 2 points; inadequately specified: 1 point; not specified: 0 points; not applicable: NA. BIAS: low risk > 70%; medium risk between 70% and 50%; high risk < 50%.

## Data Availability

Not applicable.
